# Recent advances in ultrasound-targeted nanobubbles combined with cancer immunotherapy: Mechanisms, applications, and challenges

**DOI:** 10.1016/j.fmre.2024.10.017

**Published:** 2024-11-13

**Authors:** Xueqin Chen, Lifan Xu, Chen Chen, Qizhao Huang, Jianjun Hu

**Affiliations:** a Department of Dermatology, Southwest Hospital, Army Medical University, Chongqing 400038, China; b Institute of Immunology, Army Medical University, Chongqing 400038, China; c College of Animal Science and Veterinary Medicine, Southwest Minzu University, Chengdu, Sichuan 610041, China; d Institute of Immunological Innovation and Translation, Chongqing Medical University, Chongqing 400016, China; e Department of Oncology, Southwest Hospital, Army Medical University, Chongqing 400038, China

**Keywords:** Cancer immunotherapy, Ultrasound-targeted nanobubbles, Tumor microenvironment, Drug delivery, Immune checkpoint inhibitors

## Abstract

Immunotherapy has revolutionized cancer treatment by leveraging the immune system to target tumors. However, its efficacy is often limited by the immunosuppressive tumor microenvironment and the development of resistance, leading to response rates of only 20%–30%. Ultrasound-targeted nanobubbles (UTN) combined with cancer immunotherapy present a promising solution to the limitations of current treatments. By utilizing the mechanical and biological effects of ultrasound, UTN improve drug delivery, reduce systemic toxicity, and modulate immune responses within the tumor microenvironment. Preclinical studies have shown that UTN combined with cancer immunotherapy can significantly increase the use of checkpoint inhibitors, tumor vaccines, and gene-based therapies, resulting in better tumor control. This article reviews the latest advancements, applications, and challenges of UTN combined with cancer immunotherapy, emphasizing the potential of UTN to overcome current therapeutic barriers and providing a forward-looking perspective on its translation into clinical practice.

## 1 Introduction

### 1.1 Cancer immunotherapy: Achievements and limitations

Cancer is a multifactorial disease that arises from the accumulation of genetic alterations and cellular dysregulation, and it remains one of the most challenging diseases to treat because of its complex biology and heterogeneity [1]. The mortality rate attributed to cancer has consistently increased throughout the past century. The increasing incidence of cancer and its associated fatalities in China have presented formidable challenges to the nation's health care infrastructure and societal progression [2].

**Fig. 1:**
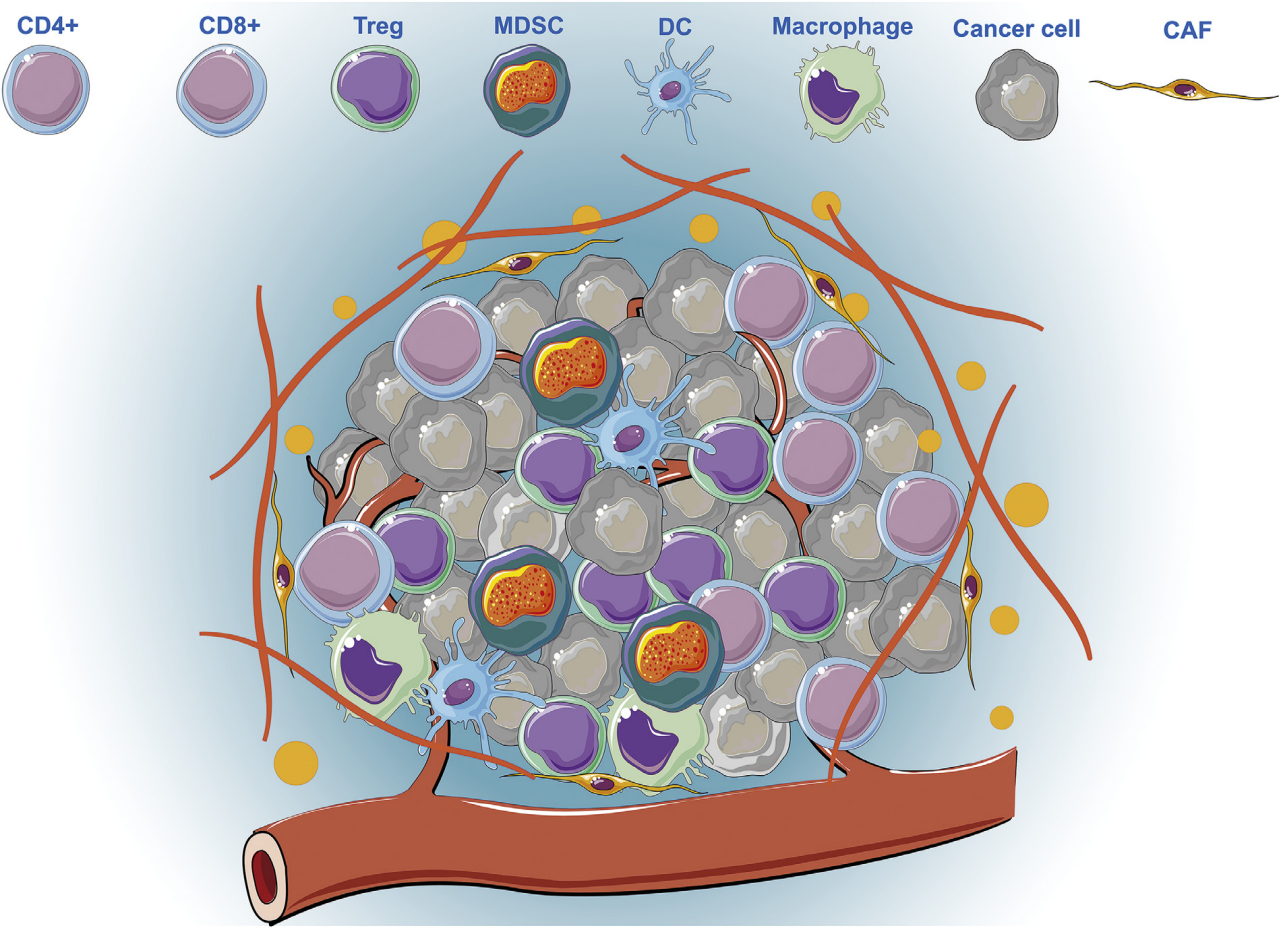
**Schematic diagram of the tumor microenvironment composition**.

Over the past decade, cancer immunotherapy has revolutionized clinical cancer treatment, particularly with the development of immune checkpoint inhibitors (ICIs) [3]. Unlike traditional methods that directly target cancer cells, immunotherapy exerts its effects by harnessing the body's immune system to repress tumor progression and reshape the tumor microenvironment (TME) [4]. The TME, which is composed of cancer cells, stromal cells, immune cells, and extracellular matrix components, plays a pivotal role in regulating tumor growth, progression, and response to therapy. Within the TME, an immunosuppressive environment often develops, characterized by the presence of regulatory T cells (Tregs), cancer-associated fibroblasts (CAF), myeloid-derived suppressor cells (MDSCs), and inhibitory cytokines such as transforming growth factor-beta (TGF-β) and interleukin-10 (IL-10), which collectively inhibit effective antitumor immune responses (Fig. 1).

This immunosuppressive TME poses a significant barrier to immunotherapy, reducing the efficacy of ICIs such as anti-CTLA-4 and anti-PD-1/PD-L1 antibodies, which have otherwise shown remarkable clinical success in various types of cancer. Despite these successes, the overall response rate for ICIs remains only 20%−30% [5]. Even among responders, durable responses are observed in only a small subset of patients, with many developing resistances or relapsing due to the ability of the TME to evade immune detection [6]. Additionally, ICIs can cause severe immune-related adverse events (irAEs) that can be life-threatening [7].

Given these limitations, there is an urgent need for complementary and combination approaches that can modify the immunosuppressive TME, increase the infiltration and activity of immune cells, and improve the delivery and efficacy of immunotherapeutic agents. Addressing the challenges of immune resistance and TME modulation and optimizing drug delivery are crucial for advancing the therapeutic potential of cancer immunotherapy.

### 1.2 Introduction to the ultrasound-targeted nanobubbles (UTN)

In recent years, UTN have emerged as a promising strategy to address these limitations in cancer immunotherapy [[8], [9], [10]]. They offer a unique mechanism for enhancing the delivery and efficacy of immunotherapeutic agents by utilizing ultrasound to promote cavitation and mechanical effects, thereby improving drug penetration and precise drug delivery [11]. Structurally, nanobubbles consisting of a gas core surrounded by a stabilizing shell can be engineered to carry a variety of therapeutic agents, including checkpoint inhibitors, tumor vaccines, and gene-based drugs [[12], [13], [14]]. This combined therapy has shown promising therapeutic prospects across various types of cancer, with significant progress in antitumor drug delivery, regulation of the tumor immune microenvironment, and sensitization to immunotherapeutic agents [[15], [16], [17]].

This review aims to provide an overview of the superiority of UTN combined with cancer immunotherapy, its mechanisms, and preclinical studies. We also discuss the challenges and opportunities for the clinical translation and implementation of UTN combined with cancer immunotherapy in cancer treatment. Based on these endeavors, we hope to shed light on the potential of UTN combined with cancer immunotherapy as a novel and effective cancer therapy.

## 2 Overview of UTN combined with cancer immunotherapy

### 2.1 Composition of nanobubbles

Nanobubbles are an emerging technology in cancer treatment, particularly in combination with ultrasound-targeted nanobubbles and cancer immunotherapy. Nanobubbles typically consist of a gas core and a shell layer [18]. The gas core is usually composed of medical gases such as oxygen, hydrogen peroxide, or perfluorinated compounds (e.g., C3F8 or SF6). These gases possess high acoustic responsiveness, enabling them to produce cavitation effects under ultrasound stimulation. The shell layer is typically made of phospholipids, polymers, proteins, or other surfactants, such as 1,2-dipalmitoyl-sn‑glycero-3-phosphocholine (DPPC) and polyethylene glycol (PEG)-modified phospholipids. These materials help improve the stability and biocompatibility of nanobubbles while also allowing the modification of their surface with targeting ligands to increase their specificity for tumor tissues [19].

**Fig. 2:**
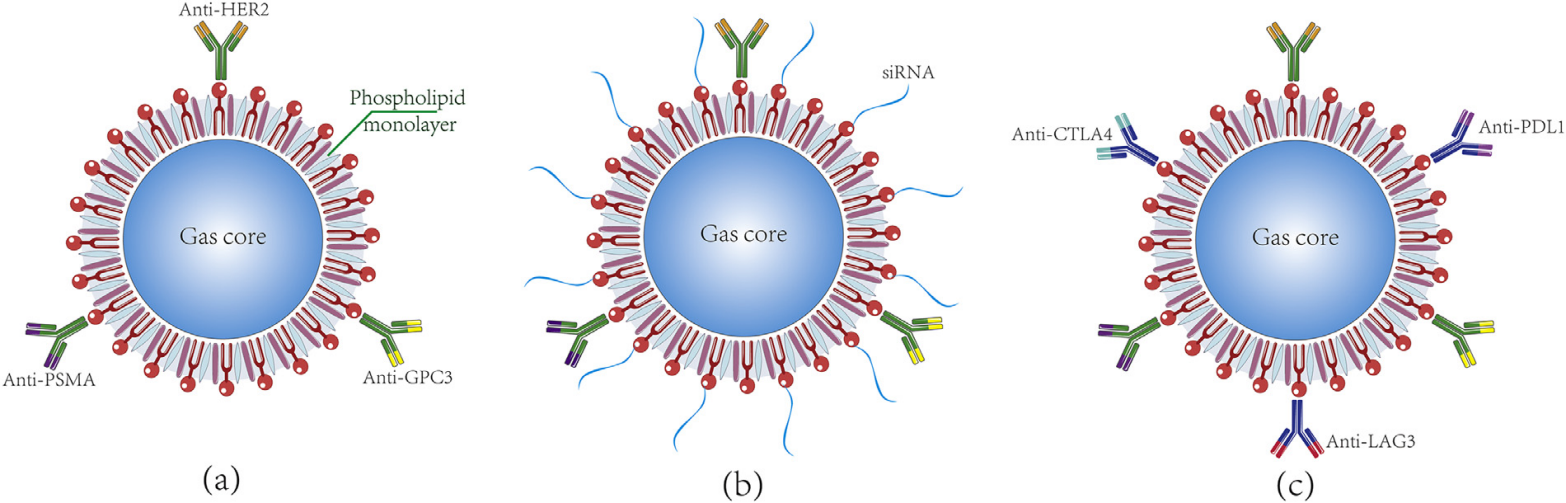
**Schematic diagram of nanobubble structures**.

To illustrate the versatility of nanobubbles in cancer treatment, Fig. 2 provides a schematic representation of nanobubble structures, highlighting three primary configurations:

(a) nanobubbles labeled with tumor-specific antibodies (e.g., HER2, GPC3, and PSMA) to enhance the targeting of tumor cells;
(b) nanobubbles modified with siRNA conjugated to targeting antibodies for gene silencing applications;
(c) nanobubbles carrying immune checkpoint blockade agents (e.g., anti-PDL1, anti-CTLA4, and anti-LAG3) functionalized with targeting antibodies to increase immunotherapeutic efficacy by modulating immune checkpoints.

### 2.2 Brief introduction of UTN combined with cancer immunotherapy

The use of nanobubbles in combination with immunotherapy was first reported in 2010 [20], when researchers showed that UTN could enhance the delivery of a plasmid encoding IL-12 to tumors in mice. Subsequent research has shown that UTN combined with cancer immunotherapy and vaccines (e.g., DNA vaccines targeting tumor-associated antigens) exert superior control over tumor growth compared with the standalone vaccine approach. Notably, the tumor microenvironment was profoundly ameliorated, enhancing the immune infiltration of CD8^+^ T cells and F4/80^+^ macrophages [[21], [22], [23], [24]]. This approach is promising for enhancing the efficacy of cancer vaccines. Since then, researchers have expanded the combined use of UTN with cancer immunotherapy using a variety of immunotherapeutic agents, including checkpoint inhibitors and gene-based drugs [[25], [26], [27]]. Preclinical studies have also shown that UTN combined with cancer immunotherapy can enhance the efficacy of these agents and improve the harsh tumor microenvironment [[28], [29], [30]]. However, the complex mechanism underlying this approach remains obscure.

## 3 Mechanisms by which UTN enhance immunotherapy

The integration of UTN in immunotherapy represents a novel and multifaceted approach to cancer treatment. This technique leverages the unique interactions between ultrasound waves and nanobubbles to increase the efficacy of immunotherapeutic agents. The underlying mechanisms are complex and involve several key processes.

### 3.1 Drug delivery enhancement

The integration of nanobubbles with immunotherapy has introduced a paradigm shift in the delivery of immunotherapeutic agents. Nanobubbles, by virtue of their size, can traverse biological barriers, enhancing the bioavailability of drugs at the tumor site. For example, the enhanced permeability and retention (EPR) effect in tumors facilitates the accumulation of nanobubbles [31,32]. A study demonstrated this effect with paclitaxel-loaded nanobubbles, leading to increased drug concentrations in tumor tissues [33]. When conjugated with immunotherapeutic agents, these nanobubbles can be directed toward the tumor microenvironment, ensuring a relatively high concentration of the drug in the vicinity of the tumor cells. Many studies have shown improved outcomes in the treatment of several tumors, including melanoma, prostate cancer, and colorectal cancer, using this approach [[34], [35], [36]].

After reaching the tumor site, upon exposure to targeted ultrasound, the nanobubbles undergo cavitation caused by oscillation and collapse, resulting in the localized release of the carrier drugs [37,38]. This ultrasound-triggered release mechanism ensures that the immunotherapeutic agents are released in a controlled manner, directly at the tumor site. The precision of this approach allows for the optimization of drug dosages, potentially enhancing the therapeutic index of immunotherapeutic agents such as immunostimulatory agents or adjuvants [39,40] immune checkpoint blockade [41,42], and even some immunomodulatory molecules delivered to dendritic cells (DCs) [43]. Simultaneously, these cavitation effects induce subtle mechanical stress and pressure changes within the tumor tissue, which not only temporarily increase vascular permeability but also directly impact the cell membranes of tumor cells. The oscillation and rupture of bubbles induced by cavitation can cause the formation of transient sonoporation in the cell membrane, making it easier for drug molecules to penetrate the cell membrane and enter tumor cells. This process significantly enhances the intracellular uptake efficiency of drugs, which ensures that more of the drug can directly target cancer cells, thereby improving drug permeability and retention within the tumor [18,44]. The cavitation effects of nanobubbles can also alter the characteristics of the tumor microenvironment. For example, the microshock waves and thermal effects generated by cavitation can disrupt the dense extracellular matrix of tumor tissue, reducing tumor interstitial pressure and subsequently improving the diffusion and distribution of drugs within the tumor tissue [45]. Additionally, ultrasound-induced cavitation can increase local blood flow, further promoting drug accumulation and penetration at the tumor site.

Nanobubbles can also achieve specific targeting of tumor cells through surface modification with specific ligands, such as antibodies, peptides, or other targeting molecules [45]. Under the influence of ultrasound, these targeted nanobubbles can localize precisely to the tumor site and undergo rupture or cavitation induced by ultrasound, thereby achieving precise drug release. This targeted release mechanism not only increases the local concentration of the drug at the tumor site but also reduces the nonspecific distribution of the drug in normal tissues, thereby lowering systemic toxicity [46,47]. As a multifunctional drug delivery platform, nanobubbles can carry multiple drugs simultaneously, resulting in a synergistic therapeutic effect. For example, nanobubbles can deliver both immune checkpoint inhibitors and tumor antigens simultaneously, which, combined with ultrasound-induced immune responses, can further increase the effectiveness of immunotherapy.

### 3.2 UTN reinforce the release of cancer cell antigens

The primary mechanism by which UTN enhance immunotherapy involves facilitating tumor antigen release, which is driven by ultrasound-induced cavitation. Cavitation occurs when UTN are exposed to ultrasound waves, leading to rapid oscillation and collapse of the nanobubbles. This collapse generates localized mechanical forces, such as high shear stress and shock waves, which exert significant physical pressure on nearby cancer cells [48]. These forces can disrupt cancer cell membranes, leading to the release of intracellular antigens [49].

The released antigens are crucial for initiating an effective immune response, as they serve as signals that are recognized by antigen-presenting cells (APCs), such as DCs. Once these antigens are captured by DCs, they are processed and presented on the cell surface in conjunction with major histocompatibility complex (MHC) molecules. This presentation activates T cells, particularly cytotoxic T lymphocytes (CTLs), which are responsible for recognizing and attacking cancer cells displaying specific antigens [50].

UTN have shown a unique ability to enhance antigen release even from tumors that are traditionally classified as “cold” tumors that are poorly infiltrated by immune cells and exhibit low levels of natural antigen release. For example, our group discussed how nanobubbles facilitate antigen release from cold tumors, such as prostate cancer, under ultrasound stimulation [13]. This increase in antigen availability can potentially convert these cold tumors into “hot” tumors, which are characterized by increased immune cell infiltration and heightened immunogenicity. Such a transformation is particularly important for improving the effectiveness of immunotherapies, as hot tumors tend to respond better to treatments that rely on immune system activation.

In conclusion, the ultrasound-induced cavitation effect of UTN plays a critical role in reinforcing the release of cancer cell antigens, promoting stronger and more effective immune recognition of tumors. By facilitating the availability of antigens, UTN enhance the overall immune response, particularly in tumors that are less responsive to conventional immunotherapy.

### 3.3 Immune modulation

UTN can modulate the immune system and enhance the recognition and killing of cancer cells. Studies have shown that nanobubbles can stimulate the production of proinflammatory cytokines such as interleukin-2 (IL-2), interleukin-6 (IL-6), tumor necrosis factor-alpha (TNF-α), and interferon-gamma (IFN-γ). These cytokines can activate immune cells and increase their cytotoxicity against cancer cells. Moreover, UTN can promote the maturation and activation of DCs, which play critical roles in initiating and regulating immune responses. DCs can capture antigens from cancer cells and present them to T cells, leading to the activation of specific CTLs that can recognize and kill cancer cells [51].

In addition, UTN can stimulate the immune system by releasing danger signals and activating immune cells. When exposed to ultrasound, nanobubbles can induce immunogenic cell death (ICD) in tumor cells. During ICD, cancer cells release not only antigens but also damage-associated molecular patterns (DAMPs), such as high-mobility group box 1 (HMGB1), calreticulin, and ATP. The released antigens and DAMPs attract and activate APCs, such as DCs and macrophages, enhancing their ability to efficiently capture and present tumor antigens and thereby activating T cells and initiating a specific antitumor immune response [[52], [53], [54]]. Wooram and colleagues developed nanobubbles capable of inducing RIPK3-independent necroptosis. This innovative approach utilizes nanobubbles that trigger immunogenic cell death in cancer via RIPK3-independent necroptosis. When combined with immune checkpoint blockade, these nanobubbles have been shown to achieve complete regression of the primary tumor in RIPK3-deficient CT26 tumor-bearing mouse models and to exhibit beneficial therapeutic effects against metastatic tumors [16]. Additionally, these processes reduce tumor-induced immunosuppressive signals, thereby enhancing the overall intensity and durability of the immune response. This process can activate effector T cells and induce the formation of memory T cells. These memory T cells persist in the body for a long term and can rapidly respond upon re-exposure to the same tumor antigens, providing continuous immune surveillance and antitumor effects [13].

Another significant aspect of UTN therapy is its abscopal effect, whereby the treatment of a local tumor site results in the systemic eradication of distant metastases and micrometastases [55]. This phenomenon occurs when the immune activation instigated by the UTN extends beyond the treated area, enabling the immune system to target and eliminate cancer cells throughout the body. By effectively treating local tumors, UTN achieve systemic, whole-body cancer therapy, offering a comprehensive approach to combating metastatic disease. Moreover, the abscopal effect of UTN can be further enhanced by combination with immunotherapy or other treatments [56].

**Fig. 3:**
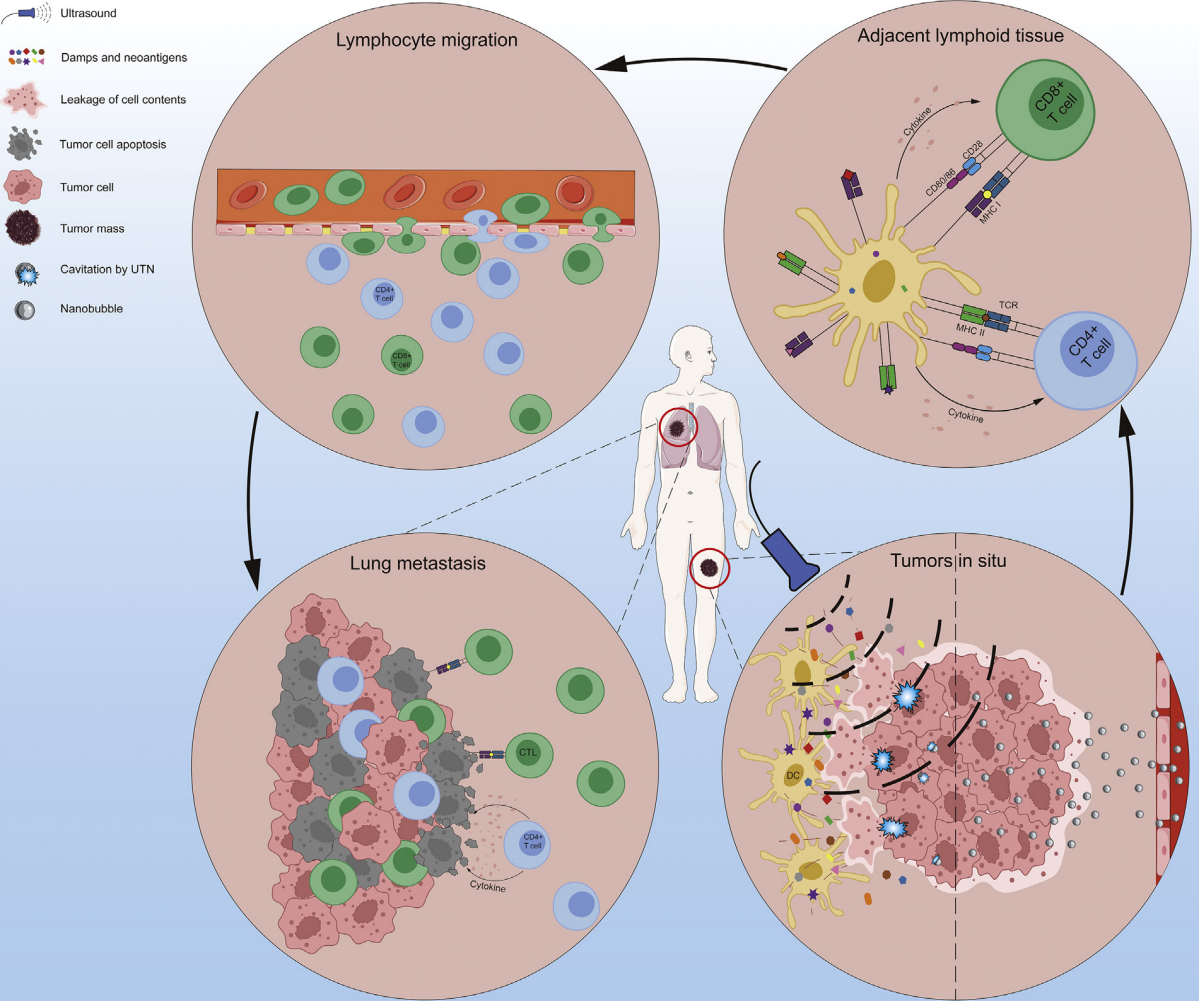
**Schematic diagram of the mechanisms by which UTN enhance immunotherapy**.

In conclusion, UTN therapy enhances immunotherapy through a combination of mechanical and biological mechanisms. These mechanisms include reinforced release of cancer cell antigens, increased drug delivery, increased immune cell infiltration, stimulation of the immune response, and induction of immunogenic cell death. The abscopal effect further amplifies the impact of UTN, facilitating the destruction of distant metastatic and micrometastatic tumor cells (Fig. 3). This approach represents a promising advancement in the field of cancer immunotherapy, offering the potential for more effective and personalized treatment strategies.

## 4 Current applications of UTN combined with immunotherapy for cancer

The integration of UTN with immunotherapy offers a transformative opportunity to increase the efficacy of conventional cancer treatments, including chemotherapy, radiotherapy, and surgery. These nanobubbles are typically composed of biocompatible materials and can be loaded with anticancer drugs, immunomodulators, or gene therapy vectors. By precisely tuning the composition of the nanobubbles, personalized treatments can be developed for different types of tumors. This customization includes modifying surface properties and encapsulating therapeutic agents to better match the tumor's biological environment, thereby enhancing therapeutic efficacy and minimizing damage to normal tissues. For example, specific targeting ligands can be attached to nanobubbles to recognize and bind to unique tumor markers. As described earlier, nanobubbles modified with a protein fused to a single-chain antibody (anti-HER2 scFv-nCytc) have been shown to enhance targeting and induce apoptosis in HER2^+^ breast tumors both in vitro and in vivo, thereby improving treatment precision [57]. Additionally, by labeling small molecules, these nanobubbles can target hepatocellular carcinoma (HCC) antigens such as GPC3 and prostate cancer antigens such as PSMA, promoting tumor regression [58,59].

### 4.1 Applications in anti-cancer immunity-related gene therapy

With the development of genetic engineering and the gradual elucidation of tumor pathogenesis, gene therapy has shown great potential in the effective treatment of tumors [60]. Recently, enormous efforts have been made to develop gene delivery nanomaterials. UTN have emerged as a powerful tool for enhancing the delivery and efficacy of gene therapy in cancer treatment. Using nanobubbles to encapsulate gene therapy agents, such as siRNA or CRISPR-Cas9 systems, allows for precise editing or silencing of tumor-promoting or inhibitory factors. In one study, ultrasound combined with nanobubbles effectively mediated CRISPR/Cas9 gene editing of the Cdh2 gene to inhibit tumor invasion and metastasis. Another study demonstrated that nanobubbles are promising US-responsive tools for siRNA delivery and are able to overcome chemoresistance in melanoma cancer cells [61,62]. This strategy is suitable for tumors with specific genetic backgrounds and can be designed based on the genetic characteristics of the patient's tumor to ensure that the therapeutic payload is delivered directly to the tumor microenvironment, thereby maximizing the treatment efficacy and minimizing off-target effects.

The UTN approach can be further tailored by incorporating specific microRNAs or other gene-modulating agents to target pathways involved in tumor progression and immune evasion, thereby enhancing both gene therapy efficacy and antitumor immune responses. For example, microRNA-122 has been shown to inhibit tumor progression, whereas microRNA-21, which is highly expressed in HCC, promotes tumor cell proliferation and migration [63]. Notably, nanobubbles loaded with microRNA-195 and microRNA-242 were shown to specifically inhibit PD-L1 expression, and combination with an anti-PD-L1 antibody could increase the infiltration of T cells and the activation of NK cells, thereby inhibiting the growth of subcutaneously transplanted hepatocellular carcinoma [31,64]. This combination not only induced the infiltration of CD8^+^ T cells into the tumor region in a canine HCC model but also reversed the immunosuppressive tumor microenvironment under ultrasound irradiation.

### 4.2 Applications in dendritic cell-based vaccination

In the context of dendritic cell-based vaccination, UTN have emerged as a powerful tool for enhancing the delivery and efficacy of vaccine components. Argenziano et al. explored the use of chitosan-shelled nanobubbles loaded with a DNA vaccine targeted to DCs. This novel approach successfully enhances antigen presentation by DCs. In addition, studies have shown that DCs treated with these nanovaccines exhibit increased maturation markers (CD80 and CD86) and higher levels of cytokine production (IL-4, IL-6, TNF-α, and IFN-γ), leading to more effective activation of CTLs [65]. In another study, DCs pulsed with nanovaccines presented increased expression of the MHC-I/SIINFEKL complex, which is crucial for T-cell activation. Furthermore, a previous study demonstrated that mice previously treated with UTN had a significantly lower incidence of tumor recurrence and better survival rates [66]. These findings suggest that UTN vaccination not only affects current tumor cells but also prepares the immune system to recognize and respond to future tumor challenges, providing ongoing protection against cancer. This method enhances the delivery and efficacy of vaccines, induces strong and durable antitumor immune responses, and provides long-term protection against cancer.

### 4.3 Applications in enhancing immune checkpoint inhibitors

UTN have demonstrated significant potential in enhancing the delivery and efficacy of ICIs, a cornerstone of modern cancer immunotherapy. The primary mechanism by which ICIs are enhanced involves the precise delivery of checkpoint-blocking antibodies directly into the tumor microenvironment. This targeted approach reduces systemic exposure and potential irAEs while maximizing therapeutic effects within the tumor [67]. Nanobubbles carrying anti-PD-L1 or anti-PD-1 antibodies can be utilized for tumors that respond well to PD-L1/PD-1 inhibitors [68]. For example, a study by Chen et al. demonstrated that PD-1 antibody-conjugated nanobubbles, when combined with targeted ultrasound, significantly increased the concentration of drugs at the tumor site and improved the inhibition of tumor growth in hepatoma tumor models [68]. This method ensures that higher doses of ICIs can be localized to tumors, potentially overcoming resistance mechanisms and improving response rates. Additionally, the combination of UTN with ICIs can lead to synergistic effects, where the mechanical action of the nanobubbles complements the biological action of the immune checkpoint blockade. This synergy can lead to ICD of tumor cells and potentially turn 'cold' tumors, which typically do not respond well to immunotherapy, into 'hot' tumors that exhibit high levels of immune cell infiltration and activity [27,69]. For patients requiring CTLA-4 inhibition, nanobubbles encapsulating CTLA-4 inhibitors can be employed. Similarly, nanobubbles can be tailored to deliver other immunotherapeutic agents, such as IL-2, IL-12, and IFN-γ, according to the specific needs of the patient, thereby enhancing the antitumor immune response.

In summary, current applications of UTN combined with immunotherapy in cancer treatment are multifaceted, ranging from enhancing anticancer immunity-related gene therapy and dendritic cell-based vaccinations to improving ICIs. Relevant reports of UTN combined with immunotherapy for cancer are listed in Table 1. These applications leverage the unique properties of UTN, such as targeted delivery and enhanced ICD, to improve the efficacy and specificity of immunotherapeutic agents. As research in this field continues to evolve, UTN have the potential to significantly advance the landscape of cancer immunotherapy.

**Table 1:** **Summary of current applications of UTN combined with immunotherapy for cancer treatment**.

Authors & year of publication	Immunotherapy methods	Cancer types	Treatment	Animal model	Mechanism
Wooram et al. (2020) [16]	Enhancing Immune Checkpoint Inhibitors	Colorectal cancer	NBs (PFP+PEGylated carboxymethyl dextran and C e6)+US+aPD-L1	RIPK3-deficient CT26 tumor-bearing mice	NBs enhance cancer immunotherapy by inducing RIPK3-dependent necrosis and boosting PD-L1 blockade efficacy.
Zhao et al. (2021) [30]	Enhancing Immune Checkpoint Inhibitors	Triple-negative breast cancer	Ce6/MET NPs-^D^PPA1+PDT+PD-L1	BALB/c mice	Enhancement of PDT effectiveness by addressing the hypoxic tumor microenvironment through the combined actions of the drugs and the nanobubbles technology
Tan et al. (2021) [27]	Enhancing Immune Checkpoint Inhibitors	HCC	sPD-1/Ce6-NBs+SDT	Male BALB/c mice	Targeted delivery of Ce6 and sPD-1 boosts UTND's anti-tumor effects, enhancing immune response and synergistic HCC immunotherapy.
Argenziano et al. (2022) [65]	DNA vaccine	HER2+ breast cancer	CD11c-targeted HER2-NBs+	BALB/c mice	They selectively transfected and activated DCs, delaying tumor growth in HER2+ breast cancer mice by triggering specific immune responses.
Yao Ma et al. (2022) [31]	Gene Therapy	HCC	miR-195 and Ce6 co-loading NBs+SDT	Male BALB/c mice	SDT-induced ICD and miR-195 enhanced PD-1/PD-L1 blockade, triggering stronger antitumor immunity.
Yun Liu et al. (2022) [70]	Enhancing Immune Checkpoint Inhibitors	HCC	ICIS+PD-L1Ab/Ce6-NBs+SDT	Balb/c mice	enhancing the body's immune response against HCC cells and inducing ICD through ROS production, triggered by ultrasound-activated Ce6.
Yun Liu et al. (2022) [64]	Gene Therapy	HCC	PD-L1 Ab/miR-424-NBs+SDT	SPF-level BALB/c mice	PD-L1 antibody and miR-424 boost T cell-mediated antitumor response, inhibiting hepatocellular carcinoma in mice via acoustic delivery.
Yezi Chen et al. (2022) [68]	Enhancing Immune Checkpoint Inhibitors	HCC	PD-L1 mAb/DOX NBs+SDT	Female BALB/c normal mice	Ultrasound-mediated PD-L1 mAb/DOX-NBs enhance tumor targeting, blocking immune tolerance and activating antitumor immunity with DOX-induced ICD.
Jianjun Hu et al. (2022) [13]	Enhancing Immune Checkpoint Inhibitors	prostate cancer,colon cancer,melanoma	NBs+anti-PD1+US	C57BL/6 mice	USNBs convert cold tumors to hot, enhancing anti-PD1 therapy, and providing long-term tumor suppression and immune memory.
Yichi Chen et al. (2023) [71]	Enhancing Immune Checkpoint Inhibitors	HCC	ICG@C3F8-R848 NBs+SDT	male C57BL/6 mice	ROS release induces apoptosis, while R848 repolarizes M2 to M1 macrophages, reversing tumor immunosuppression.
Xin Huang et al. (2024) [52]	Enhancing Immune Checkpoint Inhibitors	prostate Cancer	Ce6@aPD-L1 NBs+SDT	BALB/C-Nude male mice	NBs inhibit tumor growth and enhance immunity via SDT-induced ICD and PD-1/PD-L1 blockade, activating DC maturation and CD8+ *T* cells.

Poly(lactic-coglycolic) acid copolymer-poly(ethylene glycol) (PLGA-b-PEG), perfluoropentane (PFP), chlorin e6 (Ce6), ultrasound (US), anti-PD-L1 antibody (aPD-L1), receptor-interacting protein kinase 3 (RIPK3), NK cell-mimetic drug-based self-assembled nanohybrids (NK-DNH), oxaliplatin (OXA), 1-methyl-d-tryptophan (1-MT), ovalbumin (OVA), nanocomposite hydrogel (NC gel), metformin (MET), anti-PD-L1 peptide (DPPA-1), photodynamic therapy (PDT), sonodynamic therapy (SDT), ultrasound-targeted NB destruction (UTND), chemiluminescence resonance energy transfer (CRET)-based immunostimulatory nanoparticles (iCRET NPs), reactive oxygen species (ROS), female-specific pathogen-free (SPF), doxorubicin (DOX), ultrasound-stimulated nanobubbles (USNBs), immunogenic cell death (ICD).

## 5 Challenges and limitations for the clinical application of UTN combined with cancer immunotherapy

UTN is a promising approach in cancer treatment that has shown potential for improving the efficacy of immunotherapy. While preclinical and clinical studies have demonstrated the feasibility and safety of this approach, several challenges remain to be addressed before this approach can be widely adopted in clinical practice [72]. In this section, we discuss the challenges and limitations of the clinical application of UTN combined with cancer immunotherapy in cancer treatment.

### 5.1 Challenges

#### 5.1.1 Immunological and tumor complexity

One of the primary biological challenges lies in the complexity of the immune response and the tumor microenvironment. The immune system response to cancer is intricate and can be influenced by various factors, including the presence of immunosuppressive cells and molecules within the tumor microenvironment [73]. These factors can hinder the effectiveness of UTN combined with cancer immunotherapy. Additionally, there is heterogeneity in tumors, both between different patients and within a single tumor. This heterogeneity can lead to variable responses to therapy and the development of resistance. Therefore, the heterogeneity of tumors and individual patient responses pose significant challenges in personalizing UTN-based therapy.

#### 5.1.2 Precision delivery and stability of nanobubbles

Technical challenges primarily revolve around the delivery and stability of UTN. Ensuring the consistent and targeted delivery of nanobubbles to the tumor site is crucial for their effectiveness. This targeting requires precise control over the size, stability, and surface properties of the nanobubbles, as well as the ultrasound parameters used for their activation [18]. Stability is another critical issue, as the structural integrity of nanobubbles must be maintained during circulation to ensure effective delivery and activation at the target site [74]. Furthermore, the scalability of nanobubbles production and the reproducibility of their therapeutic effects are essential considerations for clinical translation. Advances in nanotechnology and ultrasound engineering are addressing these challenges [75], but there remains a need for standardized protocols and optimization to ensure reliable and effective treatments.

#### 5.1.3 Ethical and regulatory considerations

Ethical and regulatory considerations are paramount in the development and clinical application of any new medical technology. For UTN in cancer immunotherapy, these considerations include patient safety, informed consent, and the ethical implications of experimental treatments. Ensuring patient safety is the foremost concern, particularly in early-stage clinical trials where the risks and benefits of the new therapy may not be fully understood. Informed consent is critical, and patients must be thoroughly informed about the experimental nature of the treatment, including its potential risks and benefits. Additionally, regulatory hurdles must be overcome, as new therapies require rigorous testing and approval processes to ensure their safety and efficacy [76,77]. Navigating these ethical and regulatory landscapes is essential for the successful development and implementation of these therapies in clinical practice.

### 5.2 Limitations

#### 5.2.1 Complex mechanisms

The underlying mechanisms by which UTN enhance the efficacy of cancer immunotherapy remain highly complex and have not yet been fully elucidated. This gap in mechanistic knowledge poses a significant barrier to the refinement, optimization, and personalized application of UTN-based therapeutic strategies [45]. The behavior of nanobubbles under ultrasound activation, particularly their interaction with biological tissues and immune cells, is influenced by a multitude of variables. These include the ultrasound frequency, intensity, duration, and physicochemical properties of the nanobubbles themselves, such as size, shell composition, and gas core. Variability in these factors could lead to inconsistent therapeutic outcomes, affecting both the precision and overall efficacy of the treatment. Additionally, the lack of a standardized framework for modulating these variables hinders the reproducibility of results across different experimental settings, complicating the clinical translation of this promising modality [15,46].

#### 5.2.2 Feasibility in clinical applications

Despite the encouraging findings demonstrated in preclinical models, the clinical applicability of UTN in conjunction with cancer immunotherapy remains a significant challenge. To date, no large-scale clinical trials have been conducted to validate the safety, efficacy, and therapeutic benefit of this combined approach in human patients [18]. The scalability of UTN technology for widespread clinical use is further constrained by the technical complexities involved in the synthesis and functionalization of nanobubbles. The processes required to tailor nanobubbles for specific biological targets, including modifications to increase their biocompatibility and stability in the bloodstream, are not only intricate but also prohibitively expensive. Moreover, integrating UTN with existing cancer treatment regimens, such as immune checkpoint inhibitors or chemotherapy, introduces additional layers of complexity that need to be addressed in clinical protocols. These barriers underscore the necessity for further large-scale, controlled clinical trials to establish the viability and optimization of UTN-based cancer immunotherapy in clinical practice [78].

#### 5.2.3 Potential toxicity

The potential for adverse biological reactions, particularly immunogenicity and toxicity, represents a critical limitation of UTN therapy. The human immune system may recognize nanobubbles as foreign entities, eliciting an unintended immune response that could undermine therapeutic efficacy or lead to harmful side effects. Furthermore, residual or excessively accumulated nanobubbles could alter serum viscosity, disrupt normal physiological functions, or exacerbate immune reactions. In the context of cancer immunotherapy, where immune modulation is already delicate, the introduction of UTN adds an additional layer of complexity. For example, when used in combination with immune checkpoint inhibitors, such as PD-1 or CTLA-4 inhibitors, UTN might excessively stimulate the immune system, potentially leading to systemic immune-related adverse events. These adverse events include but are not limited to cytokine release syndrome, autoimmune reactions, or tissue damage. Consequently, careful monitoring and thorough preclinical evaluations are necessary to mitigate these risks, ensuring the safe integration of UTN in cancer immunotherapy protocols [30].

## 6 Conclusion

UTN combined with cancer immunotherapy represent an innovative and promising approach for overcoming some of the key limitations of current cancer treatments, particularly with regard to enhancing drug delivery and modulating the tumor microenvironment. By leveraging the mechanical and biological effects of ultrasound, UTN enhances the precision and efficacy of immunotherapeutic agents, offering potential solutions to challenges such as immune resistance and tumor heterogeneity. Preclinical studies have demonstrated the capacity of UTN to amplify the therapeutic effects of checkpoint inhibitors, tumor vaccines, and gene-based therapies, paving the way for more effective tumor control.

However, despite these advances, significant challenges remain in the clinical translation of this technology. The mechanistic complexity of UTN, particularly in terms of their interactions with biological tissues and immune cells under ultrasound activation, presents obstacles to the consistent and reproducible application of this therapy. The current lack of large-scale clinical trials further complicates its adoption, because the safety, efficacy, and long-term outcomes need to be rigorously validated in human populations. Moreover, issues of potential toxicity, immunogenicity, and immune-related adverse events must be carefully addressed. The immune system response to UTN, particularly when used in conjunction with potent immunotherapies such as PD-1 and CTLA-4 inhibitors, requires meticulous preclinical assessment and clinical monitoring to prevent harmful side effects.

In summary, while UTN hold great potential for enhancing cancer immunotherapy, their path to clinical implementation will require overcoming substantial biological, technical, and regulatory challenges. Continued research into the mechanisms of UTN action, coupled with the development of standardized protocols and large-scale clinical validation, will be critical for advancing this promising therapeutic modality into mainstream cancer treatment.

## Author contributions

J.H. and X.C. conceived the manuscript. J.H. wrote the original draft. Q.H. and L.X. reviewed the draft with detailed comments. X.C. and C.C. revised the manuscript.

## Availability of data and materials

All the data generated during this study are included in this published article and its supplementary files.

## Declaration of competing interest

The authors declare that they have no conflicts of interest in this work.
